# T Cell Antifungal Immunity and the Role of C-Type Lectin Receptors

**DOI:** 10.1016/j.it.2019.11.007

**Published:** 2020-01

**Authors:** Emily A. Speakman, Ivy M. Dambuza, Fabián Salazar, Gordon D. Brown

**Affiliations:** 1 Medical Research Council (MRC) Centre for Medical Mycology, University of Exeter, Exeter EX4 4QD, UK

**Keywords:** adaptive T cell immunity, antifungal immunity, C-type lectin receptors, fungal pathogens, mycobiome

## Abstract

Fungi can cause disease in humans, from mucocutaneous to life-threatening systemic infections. Initiation of antifungal immunity involves fungal recognition by pattern recognition receptors such as C-type lectin receptors (CLRs). These germline-encoded receptors trigger a multitude of innate responses including phagocytosis, fungal killing, and antigen presentation which can also shape the development of adaptive immunity. Recently, studies have shed light on how CLRs directly or indirectly modulate lymphocyte function. Moreover, CLR-mediated recognition of commensal fungi maintains homeostasis and prevents invasion from opportunistic commensals. We present an overview of current knowledge of antifungal T cell immune responses, with emphasis on the role of C-type lectins, and discuss how these receptors modulate these responses at different levels.

## Highlights

CLRs are essential pattern recognition receptors involved in fungal recognition and initiation of protective antifungal immunity.
CLRs promote the differentiation of mammalian T helper cell subsets essential for the control of systemic (Th1) and mucosal (Th17) fungal infections.
CLRs are involved in antigen presentation, the expression of co-stimulatory molecules, and cytokine secretion; therefore, they can regulate lymphocyte function and adaptive immune responses at different levels.
Fungal morphological changes, such as the transition from yeast to hyphae in *Candida albicans* during tissue invasion, affects recognition by CLRs and impacts on adaptive immune responses.
CLRs recognize the fungal component of the microbiome that can influence T cell responses during infection at intestinal and peripheral sites.

## Immunity to Fungi

**Figure 1:**
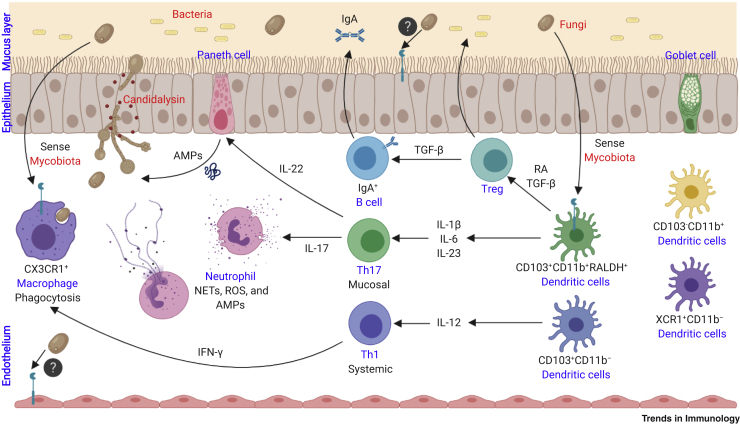
Central Role of Mammalian Dendritic Cells (DCs) in Innate and Adaptive Immunity to Fungi. Innate immune responses to fungi are mainly orchestrated by phagocytes and the epithelium. Toxins secreted by fungi such as candidalysin can directly damage epithelial membranes and trigger a danger-response signaling pathway that activates epithelial immunity [1]. Paneth cells produce molecules with antimicrobial activity as well as cytokines that can recruit other immune cells to contribute to fungal clearance [5, 6, 7]. Phagocytes such as macrophages are activated by interferon (IFN)-γ produced by T helper (Th)1 cells, and invariant natural killer T (iNKT) cells (not shown) can also play a pivotal role during superficial systemic infections [4]. The chemokine receptor CX3CR1^+^ mononuclear phagocytes express C-type lectin receptors (CLRs) that recognize the fungal component of the microbiota and promote antifungal immunity [109]. Neutrophils are activated by interleukin (IL)-17 produced by Th17 and γδ T cells (not shown), and are important at mucosal sites [9]. Th17 cells also produce IL-22 that promotes secretion of antimicrobial peptides (AMPs) such as β-defensins by epithelial cells [37]. CLR expression on DCs is important for sensing fungi and activating antigen-specific CD4^+^ T cell differentiation. Diverse subsets of DCs are present at different anatomical tissue sites and their CLR expression patterns as well as their roles during fungal infections are emerging. For instance, CD103^+^CD11b^+^RALDH^+^ DCs regulate gut mycobiota by promoting Th17 immunity, Foxp3^+^ Treg induction, and IgA production [16]. By contrast, CD103^+^CD11b^−^ DCs can support Th1 immunity via IL-12 production [17, 18, 19]. Questions (?) remain regarding CLR expression in the nonhematopoietic component (i.e., epithelial and endothelial cells) of different tissues. This becomes relevant at mucosal sites where epithelial cells provide a crucial first line of defense against pathogens, whereas endothelial cells may play a pivotal role during systemic infections. Abbreviations: NET, neutrophil extracellular trap; RA, retinoic acid; ROS, reactive oxygen species; TGF-β, transforming growth factor β; Treg, regulatory T cell.

Fungi are abundant in the environment and we are in constant contact with these organisms, from inhaled spores of *Aspergillus* spp. to **commensal fungi** (see Glossary) such as *Candida* spp. that become **opportunistic pathogens** under particular conditions. Of note, toxins secreted by fungi such as candidalysin can directly damage epithelial membranes and trigger a danger response signaling pathway that activates epithelial immunity [1]. Despite high levels of exposure, the incidence of lethal fungal infections in humans is relatively low, mainly because of a highly sophisticated immune system. This is underscored by the increased susceptibility to fungal infections that is associated with loss of immune function, as observed in individuals with HIV/AIDS who present with a range of invasive and noninvasive fungal infections such as cryptococcal meningitis and oropharyngeal candidiasis (OPC), respectively [2]. Systemic infections are relatively rare but have high mortality rates that often exceed 50%, depending on underlying conditions [3]. Successful antifungal immunity relies on both the innate and adaptive immune systems. Innate immunity constitutes the first line of defense, which includes physical barriers such as skin and mucosa, antimicrobial peptides (AMPs), the complement system, and cell-mediated protection. Effector mechanisms of innate immunity are performed by phagocytic cells such as neutrophils, macrophages, and monocytes, which mediate several protective mechanisms including phagocytosis and the production of reactive oxygen species (ROS) and hydrolytic enzymes that can directly kill fungal pathogens, as well as releasing inflammatory mediators such as cytokines [4]. Epithelial cells can also promote protection against fungi by secreting AMPs that have fungicidal and fungistatic activity through permeabilization of the cell wall and by promoting ROS production and mitochondrial dysfunction [5, 6, 7, 8] (Figure 1).

**Figure 2:**
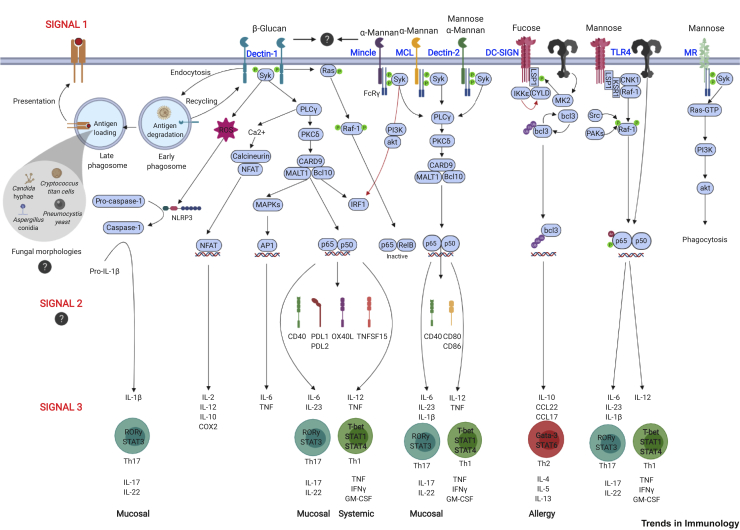
Key Figure. Signaling via C-Type Lectin Receptors (CLRs) Can Influence Antifungal CD4^+^ T Cell Immunity CLR intracellular signaling pathways promote dendritic cell (DC) maturation and migration to the draining lymph nodes where they activate naïve T cells. Dectin-1 signals through intracellular immunoreceptor tyrosine-based activation motif (ITAM) domains, whereas Dectin-2, MCL, and Mincle recruit FcRγ chains which promote the recruitment of Syk and subsequent formation of the caspase recruitment domain family member 9/mucosa-associated lymphoid tissue lymphoma translocation protein 1/B cell lymphoma 10 (CARD9/MALT1/Bcl10) complex. Dectin-1 also induces other signaling molecules such as Ras/Raf-1, Ca^2+^/calcineurin/NFAT, and NLRP3/caspase-1 in a Syk-dependent and -independent manner. These signaling cascades activate transcription factors such as nuclear factor (NF)-κB, nuclear factor of activated T-cells (NFAT), and AP1 that promote inflammation [9]. Synergistic and/or antagonistic interactions could arise from simultaneous engagement of several CLRs. For instance, Mincle can interfere with Dectin-1-mediated interleukin (IL)-12 production and type 1 T helper cell (Th1) polarization by targeting interferon regulatory factor 1 (IRF1) for degradation through the PI3K/Akt-dependent activation of the E3 ubiquitin ligase Mdm2 (red arrow) [74]. CLR signaling can modulate three signals required for T cell activation. Signal 1 (antigen presentation): antigenic peptides are presented on MHC-II molecules for recognition and priming of naïve CD4^+^ T cells. Signal 2 (co-stimulation): provides support to peptide–MHC/T cell receptor (TCR) activation. CLRs such as Dectin-1, Dectin-2, and Mincle activate the induction of co-stimulatory molecules including CD40 and CD86 [115, 116, 117]). Signal 3 (cytokines and chemokines): CLR signaling has been directly linked to the release of chemokines and cytokines that promote CD4^+^ T cell differentiation into specific T helper cell subsets including Th1, Th2, Th9, Th17, and regulatory T cells (Tregs). Dectin-1 can support both Th1 and Th17 responses through the induction of IL-12 and IL-23 that are required for systemic and mucosal immunity, respectively. Dectin-2 preferentially promotes Th17 responses via the induction of IL-23, IL-6, and IL-1β [65,66]. The induction of these T helper cells produces a second wave of chemokines and cytokines that promote the maturation, activation, and recruitment of other immune cells such as neutrophils and monocytes, resulting in fungal killing and clearance. Outstanding questions (?) remain: how do different fungal morphologies influence antigen processing and presentation? In the skin, filamentous forms of *Candida albicans* can induce a Th1 response, whereas yeast forms preferentially promote a Th17 response through Dectin-1 engagement on Langerhans cells [90]. Identification of specific immunodominant epitopes from fungal pathogens could help to better understand this process. Are additional co-stimulatory or coinhibitory molecules regulated by CLRs? How is the ligation of multiple CLRs integrated to achieve an appropriate adaptive immune response? Abbreviation: GM-CSF, granulocyte-macrophage colony-stimulating factor; IFN, interferon; P, phosphorylation; ROS, reactive oxygen species; TNF, tumor necrosis factor.

Central to initiation of protective antifungal immunity are members of the CLR superfamily which include Dectin-1 (CLEC7A), Dectin-2 (CLEC4N), macrophage C-type lectin (MCL, CLEC4D), macrophage-inducible C-type lectin (Mincle, CLEC4E), mannose receptor (MR, CD206), dendritic cell-specific intercellular adhesion molecule-3-grabbing nonintegrin (DC-SIGN, CD209), and melanin-sensing C-type lectin (MelLec, CLEC1A) [9]. CLRs are primarily expressed on cells of myeloid origin [10], but some are expressed by nonhematopoietic cells such as epithelial and endothelial cells [11]. CLRs contain at least one C-type lectin-like domain (CTLD) that is classically associated with the recognition of fungal carbohydrates such as β-glucans, mannan, and chitin that are present within the cell wall [10], but can also recognize other components such as melanin [11]. Following ligand recognition, most CLRs trigger intracellular pathways involving Syk kinase and caspase recruitment domain-containing protein 9 (CARD9), or Raf-1 (Figure 2, Key Figure). The signaling pathways of other CLRs (including MelLec and the MR) are not well defined. The CARD9 pathway is essential because defects in this pathway lead to severe systemic infections in both mice and humans [12,13]. In addition, CARD9 plays an essential role in the maintenance of gut **homeostasis** with commensal organisms and prevents tissue invasion by opportunistic fungi (see below). Recognition of fungi by CLRs activates innate host defense mechanisms which promote fungal killing but also couple with activation of the adaptive immune system. This is carried out by dendritic cells (DCs) which, upon activation, upregulate antigen-presentation molecules including **major histocompatibility complex (MHC-I/II)** molecules, enhance the expression of **co-stimulatory molecules**, and release cytokines and chemokines. This review highlights the influence of CLRs in promoting antifungal T cell immunity, with specific focus on recent advances in CD4^+^ T cell immunity. The emerging role of the mycobiome in antifungal T cell immunity will also be discussed.

## T Cell Antifungal Immunity

CD4^+^ T cells are generally considered to play a role in both the resolution and worsening of superficial or invasive fungal infections. For example, data from mouse models have indicated that T helper (Th)1 and Th17 cells are usually important for controlling fungal infections, whereas regulatory T cells (Tregs) are nonprotective. Other subsets such as Th2 and Th9 cells, as well as CD8^+^ T cells, have also been implicated in antifungal immunity (Box 1 for details). Of note, recent *in vitro* data using human leukocytes suggested that double-positive CD4^hi^CD8^lo^ T cells were involved in the control of *Candida albicans* during infection, whereas persistent infection was associated with increased CD4^lo^CD8^hi^ T cells [14]. The generation of different T cell subsets relies on activities of antigen-presenting cells (APCs) which recognize fungi through CLRs. Most of what is known about the role of CLRs in adaptive T cell immunity is derived from *in vitro* and *in vivo* studies that investigate the cytokine milieu secreted by DCs or macrophages following receptor engagement with fungi in wild-type (WT) or gene-deficient mice, CLR crosslinking with antibodies, or CLR interaction with purified ligand **agonists**/**antagonists**. For instance, numerous *in vitro* and *in vivo* studies have shown that recognition of *Candida* spp. by CLRs including Dectin-1 results in interleukin (IL)-12 production by DCs and macrophages (which promotes Th1 cell immunity that is required for protection against systemic fungal infections), and in secretion of IL-1β, IL-6, and IL-23 (which promote Th17 immunity that is crucial for restraining oropharyngeal and mucocutaneous fungal infections) [15] (Figure 1). Of note, CD103^+^CD11b^+^RALDH^+^ DCs have been found to regulate gut mycobiota by promoting Th17 immunity, Foxp3^+^ Treg induction, and IgA production in mice [16]. By contrast, CD103^+^CD11b^−^ murine DCs can support Th1 immunity via IL-12 production [17, 18, 19]. Other CLRs have been linked with the regulation of adaptive T cell immunity in the context of viral and bacterial infections (Box 2). How activation of the CLR–Syk–CARD9 pathway regulates other aspects of **APC maturation** such as the expression of co-stimulatory molecules that are necessary for T cell differentiation, survival, and memory development remains unclear (Figure 2).

Box 1 **Roles of Th2, Th9, and CD8+ T cells in Antifungal Immunity**

CLRs have been implicated in Th2-mediated fungal allergic responses. For instance, in mice, following repeated lung exposure to *A. fumigatus*, CD4^+^ T cells produce IL-4, IL-5, and IL-13 in a Dectin-1-dependent fashion (*Clec7a*^*−/−*^ mice) [119]. Moreover, intratracheal administration of *Aspergillus versicolor* spores in mice exacerbated house dust mite-induced allergic asthma through the production of IL-4 and IL-13 [120]. Although these studies support a pathological role for Th2 immunity, there are also data demonstrating a protective role for Th2 cells during fungal infections. In mice infected with pulmonary *Pneumocystis murina*, IL-13-induced alternatively activated macrophages were shown to be more fungicidal than noninduced macrophages [26]. Furthermore, in IL-4-deficient mice, intragastric challenge with *C. albicans* led to increased susceptibility to disease and failure to produce a Th1 response relative to WT mice [121].

The role of CLRs in Th9 differentiation is poorly understood and knowledge about their function in fungal infections is still emerging. During fungal infection in mice, Th9 cells have been associated with failure to clear fungal pathogens while promoting fungal asthma. For instance, IL-9 KO mice intratracheally infected with *Pneumocystis* exhibit increased numbers of Th17 cells, augmented production of IL-17A and IL-23, and decreased fungal burdens in the lung relative to WT mice [122]. In addition, following chronic *A. fumigatus* allergen exposure in mice, high numbers of Th9 cells were shown to correlate with increased severity of chronic airway hyper-reactivity relative to controls [123]. Recently, in experimental leaky-gut mouse models, *Candida*-driven IL-9 production in the gut was reported to contribute to loss of barrier integrity, fungal dissemination, and inflammation relative to controls [124]. In these same mouse models, IL-9 deficiency promoted gut dysbiosis relative to WT mice, suggesting that the functions of IL-9 might also involve the regulation of the microbiota [124].

The contribution of CLRs in CD8^+^ T cell function is inferred from *in vitro* experiments showing that bone marrow-derived DC activation by curdlan promotes differentiation of naïve CD8^+^ T cells into cytotoxic T cells *in vitro* [72]. Moreover, in this study, the crosspresentation pathway involved in MHC-I loading of fungal antigens derived from direct ingestion or uptake of apoptotic cell-associated fungal antigens was reported to be mediated by Syk/CARD9 signaling [72]. Studies in mice lacking CD4^+^ T cells have supported the role of CD8^+^ T cells in antifungal immunity. For instance, following oral challenge with *C. albicans* in mice, IL-17 from CD8^+^ T cells mediated protection against fungal infection in the absence of T and B cells (*Rag1*^−/−^ mice) [50]. In addition, following intratracheal inoculation with *C. neoformans*, depletion of CD8^+^ T cells in mice lacking CD4^+^ T cells (via monoclonal antibody administration) resulted in failure to control lung fungal growth and systemic dissemination relative to control mice [125]. Moreover, subcutaneous vaccination in mice with antigen-specific memory CD8^+^ T cells producing IL-17 has been reported to promote resistance to pulmonary *Blastomyces dermatitidis* and *H. capsulatum* in the absence of CD4^+^ T cells (antibody-mediated depletion) relative to control mice [126].

Box 2 **CLRs in Adaptive Immunity during Viral and Bacterial Infections**

CLRs have also been linked to the regulation of adaptive immunity in the context of viral and bacterial infections, including dendritic cell natural killer lectin group receptor 1 (DNGR-1, Clec9a), DEC-205 (CD205), Clec5a, dendritic cell immunoreceptor (DCIR) (CLEC4a), and dendritic cell immune-activating receptor (DCAR) (Clec4b1) [68,127]. For example, relative to controls, using Clec9a blocking antibodies in mice *in vitro* and *in vivo*, DNGR-1 was shown to promote crosspresentation (a process in which dead-cell-associated extracellular antigens are taken up, processed, and presented through MHC-I molecules to CD8^+^ T cells to induce adaptive immune responses) [128]. In addition, in mice, upon systemic infection with vaccinia virus or herpes simplex virus-1 (HSV-1), DNGR-1^+^ DCs were found to be essential for animal protection because lack of DNGR1 blocked crosspresentation of these viral antigens (as evidenced by adoptive transfer experiments and the use of DNGR-1-deficient mice) [129,130]. Furthermore, following systemic HSV-1 injection in mice, DNGR-1 was shown to promote the development of TFH cells relative to controls [131]. In addition, the recognition of mycobacterial glycolipids by the FcRγ-coupled activating receptor DCAR *in vitro* can mediate the production of monocyte chemoattractant protein 1 (MCP-1) and promote Th1 immunity against mycobacteria *in vivo* in mice [132]. Lastly, using DCIR-deficient mice, DCIR signaling – a CLR bearing an inhibitory ITIM signaling motif – was shown to sustain type-I IFN signaling in DCs and control Th1 differentiation during mycobacterial infection relative to WT mice [133]. Owing to their contribution to innate and adaptive immunity, CLRs have been used to increase the effectiveness of vaccines via approaches that involve antigen coupling to molecules (antibodies or ligands) targeting CLRs on DCs. Targeting Epstein–Barr virus (EBV) nuclear antigen 1 to DEC-205 on human PBMCs (antibody-mediated), for example, led to expansion of antigen-specific CD4^+^ and CD8^+^ memory T cells that suppressed virus-infected B cells *in vitro* [134]. Conversely, blocking Clec5a with monoclonal antibodies, before infection with Dengue or Japanese encephalitis viruses in mice, reduced the secretion of proinflammatory cytokines such as TNF and IL-6, as well as the activation of NOD-, LRR-, and pyrin domain-containing protein 3 (NLRP3) inflammasomes in mice; this restored homeostasis and led to resolution of infection, suggesting that blocking of Clec5a might potentially alleviate tissue damage and protect against some viral infections [135,136]. Of note, Clec5a also modulates innate immunity in mice in response to bacterial infections *in vivo*, including *Listeria monocytogenes* and *S. aureus*, through the activation of macrophages, neutrophils, and γδ T cell effector functions [137].

### Th1 Helper Cells

The importance of Th1 immunity in antifungal defense mechanisms has been described in both mice and humans. Th1 cells secrete interferon (IFN)-γ, granulocyte-macrophage colony-stimulating factor (GM-CSF), and tumor necrosis factor (TNF) that affect phagocyte maturation and killing ability as well as APC function (Figure 1). TNF synergy with IFN-γ induces macrophage ROS production *in vitro* which is thought to contribute to *in vivo* growth arrest of intracellular fungal pathogens including *Histoplasma capsulatum* and *Coccidioides immitis* [20,21]. Furthermore, IFN-γ promotes phagocytosis, upregulation of MHC-II molecules, and antigen-presentation by APCs [22]. In humans, deficiencies in receptors for either IL-12 or IFN-γ have been associated with increased susceptibility to coccidioidomycosis and histoplasmosis [23, 24, 25]. Notably, IFN-γ immunotherapy has been shown to improve the outcome of patients with aspergillosis, cryptococcosis, or coccidioidomycosis [26]. In addition, IFN-γ immunotherapy has been associated with faster clearance of *Cryptococcus neoformans* from the cerebrospinal fluid of HIV-1-infected AIDS patients; it also results in decreased incidence of infection or severity of invasive *C. albicans* infection in patients with chronic granulomatous disease (CGD) relative to untreated controls [27,28]. GM-CSF sequesters the micronutrient zinc via upregulation of zinc exporters and was shown *in vitro* and *in vivo* to enhance macrophage ROS production and to limit intracellular yeast survival in mice [29]. Moreover, following pulmonary challenge with *Aspergillus fumigatus*, GM-CSF receptor (R)-deficient (*Csf2rb*^*−/−*^ knockout, KO) mice have shown reduced macrophage recruitment to the lungs and impaired fungicidal activity, resulting in lower survival rates compared with WT mice [30]. Moreover, patients with congenital **pulmonary alveolar proteinosis**, stemming from mutations in GM-CSFR, are more susceptible to infections with fungi including *Aspergillus* spp. and *Cryptococcus* spp. [31,32]. It is worth mentioning that some of these deficiencies are not restricted to T cells and may affect adaptive immunity in an indirect manner. For instance, although Th1 and Th2 cytokines are reduced in GM-CSF KO mice following pulmonary challenge with *C. neoformans* relative to controls, macrophage recruitment is also reduced [33]. Of note, GM-CSF production is not restricted to Th1 cells; in fact, Th17 cells from patients with inflammatory conditions including **inflammatory bowel disease (IBD)** and multiple sclerosis (MS) have been shown to produce GM-CSF *ex vivo* [34]. Others have proposed that GM-CSF-producing CD4^+^ T cells constitute a distinct subset that is regulated by STAT5 and is important in neuroinflammation [35,36]. Specifically, loss of STAT5 in CD4^+^ T cells resulted in decreased development of experimental autoimmune encephalomyelitis (EAE), a mouse model of MS, relative to WT CD4^+^ T cells that produce GM-CSF normally [35]. This was also consistent with a GM-CSF-producing CD4^+^ T cell subset in humans [36]. Thus, for the purpose of immunotherapy and vaccine design, the inherent plasticity of CD4^+^ T helper cell subsets will require careful analysis, not only in autoimmune conditions but also in the context of fungal infections.

### Th17 Helper Cells

The pivotal role of Th17 cells in antifungal immunity in mammals is well documented. Th17 cells produce cytokines, including IL-17A, IL-17F, and IL-22, that promote neutrophil trafficking, fungicidal activity, and are involved in the induction of AMPs such as S100A7, S100A8, S100A9, and β-defensins from epithelial cells and keratinocytes that defend against fungal overgrowth [37] (Figure 1). Th17 cells differentiate in response to transforming growth factor (TGF)-β and IL-6 in mice (IL-1β can substitute for TGF-β in humans), and IL-23 is crucial for the maintenance and expansion of these cells [38]. In addition, B cell-derived IL-6 can contribute to antifungal Th17 immunity following infection with *C. albicans* [39]. Th17 cells are essential for preventing mucosal fungal infections such as OPC, chronic mucocutaneous candidiasis (CMC), and *Malassezia* skin infections [40]. In mice, Th17 cells have also been shown to support the production of IgA, an immunoglobulin crucial for protection and homeostasis at mucosal surfaces [41,42]. In fact, the plasticity of Th17 cells to convert into T follicular helper (TFH) cells may be essential to induce the development of IgA-producing B cells [41]. Thus, *Candida*-specific Th17 cells may be important during intestinal homeostasis with fungi via their capacity to convert to TFH cells. In humans, deficiency in the IL-17/IL-17R axis and in signaling components, including genetic defects in STAT1 and STAT3 (**hyper-IgE syndrome**), results in increased susceptibility to CMC [43]. Similarly, patients with autoimmune polyendocrinopathy with candidiasis and ectodermal dystrophy (APECED, or autoimmune polyendocrine syndrome 1) that produce **autoantibodies** against Th17-derived cytokines such as IL-17A, IL-17F, and IL-22 also show increased susceptibility to CMC relative to healthy controls [44,45]. These findings have been validated in mouse models, where deficiencies in the IL-17/IL-17R axis also lead to increased susceptibility relative to WT mice to mucosal *Candida* infections, including CMC, OPC, and epicutaneous candidiasis, that are associated with reduced neutrophil recruitment [46, 47, 48]. However, contrary to these results, the resulting increased susceptibility to OPC was recently suggested to be largely driven by a strong defect in the production of AMPs such as β-defensins, given that neutrophil recruitment was not impaired in IL-17A KO mice (*Il17ra*^*−/−*^ ) [49].

The therapeutic potential of Th17 cells has been explored. Adoptive transfer of WT Th17 cells into **Rag1 KO mice (*Rag1***^***−/−***^**)** was shown to promote fungal clearance during OPC [50]. In addition, WT mice rechallenged orally with *C. albicans* induced a protective antigen-specific Th17 response, suggesting that Th17 immunity is essential for long-term protection against OPC [50]. Moreover, recent data using human peripheral blood mononuclear cells (PBMCs) stimulated *in vitro* with *A. fumigatus* or *C. albicans* showed that a majority of CD4^+^ T cells were responsive to both fungal species [51]. In addition, studies in mice have also shown that the gut mycobiome may be involved in providing protection against other pathogens at distal sites [51]. For instance, human Th17 cells induced in response to gut *C. albicans*, can be expanded in the lung by crossreactive airborne *A. fumigatus* [51]. However, this poses a more fundamental question about Th17 cell biology: namely, the T cell receptor (TCR) repertoire utilized, given the promiscuity of crossreactivity with other fungal peptide antigens [51]. Moreover, Th17 cells are notorious for contributing to autoimmune disease; thus the question remains as to whether *C. albicans* is always required for induction of a Th17 response. Therefore, given that only a select few fungal pathogens can induce Th17 immunity, can we sufficiently delineate protective versus pathogenic antifungal Th17 immunity? In addition, can we define the DCs and CLRs that are required for Th17 cell differentiation?

### Role of CLRs in Th1 and Th17 Responses

The importance of CLRs in the development of Th1 and Th17 responses has been gleaned through the use of KO animals and individuals with genetic alterations in these receptors (Table 1). For instance, following systemic challenge of mice with *Candida* spp., the absence of Dectin-1 (*Clec7a*^*−/−*^) was associated with inappropriate activation and death of antigen-specific CD4^+^ T cells in the gut and reduced expression of cytokines such as TNF and IL-6 relative to WT mice [52]. Similarly, loss of Dectin-1 in mice (*Clec7a*^*−/−*^) has been shown to have a negative impact on Th1 and Th17 cell differentiation during pulmonary infection with *A. fumigatus* [53]. In humans, genetic polymorphisms in *Dectin-1* have been reported to lead to increased susceptibility to fungal disease in several patient groups. For example, patients with hematological malignancies undergoing hematopoietic stem cell transplantation (HSCT) and with an early stop codon polymorphism in Dectin-1 (Y238X) were associated with susceptibility to invasive aspergillosis through impairment of both recipient- and donor-dependent mechanisms of antifungal immunity, and exhibited a higher risk of extrapulmonary invasive *Aspergillus* infection compared with those harboring WT Dectin-1 [54]. The risk was further increased if both recipient and donor individuals with the Dectin-1 Y238X polymorphism. Analysis of PBMCs from these patients revealed decreased production of IFN-γ, IL-10, IL-1β, IL-6, and IL-17A relative to WT Dectin-1 controls, suggestive of defective Th1 and Th17 cell function [54]. Furthermore, patients with polymorphisms in Dectin-1 (including patients with hematological malignancies undergoing HSCT) displayed increased oral and gastrointestinal colonization with *C. albicans* and increased incidence of recurrent vulvovaginal candidiasis relative to WT Dectin-1 controls [55,56]. Moreover, PBMCs (e.g., monocytes, macrophages, Th17 cells) from these patients also showed defects in the production of IL-1β, IL-17, TNF, and IL-6 [55,56].

**Table 1:** The Impact of CLR Deficiency on Fungus-Induced T Cell Responses and Disease Pathology from Human and Mouse Studies^a^

Agent	CLR impairment	Cytokine effect	Pathology	Refs
Mucosal infections				
*C. albicans*	hDectin-1 SNPs	↓ TNF, IL-6, and IL-17	↑ Risk of recurrent VVC and CMC	[55,56]
*C. neoformans*	mDectin-2 KO	↑ IL-4, IL-5, and IL-13	↑ Pulmonary CFUs	[55,138]
	mMR KO	↓ CD4^+^ T cell proliferation	↑ Pulmonary CFUs	[63,138]
*P. murina*	mMincle KO	↑ TNF, IL-6, and IL-1Ra	↑ Pulmonary CFUs	[63,139]

Systemic infections				
*C. albicans*	mDectin-1 KO	↓ CD4^+^ T cells in the mLN	↑ Kidney and GI CFUs	[52]
	mDectin-2 KO	↓ IL-6, IL-1β, and IL-23	↑ Kidney CFUs	[52,66]
	hDectin-1 SNPs	↓ IL-1β	↑ Risk of systemic candidiasis	[56,66]
	hMR inhibitor	↓ IL-17A		[64]
	mMincle KO	↑ TNF	↑ Kidney CFUs	[64,140]
*C. glabrata*	mDectin-1 KO	↓ TNF, IL-6, IFN-γ, and IL-17	↑ Kidney and liver CFUs	[67,140]
	mDectin-2 KO	↓ TNF and IL-17	↑ Kidney and liver CFUs	[66,67]
*Candida krusei*	mDectin-1 KO	↓ TNF, IL-6, IFN-γ, and IL-17	↑ Kidney and liver CFUs	[66,141]
*A. fumigatus*	hDectin-1 SNPs	↓ IFN-γ, IL-10, IL-1β, IL-6, and IL-17A	↑ Risk of extrapulmonary IA	[54,141]

a Key and abbreviations: ↑, increased; ↓, decreased; CFUs, colony-forming units; CLR, C-type lectin receptor; CMC, chronic mucocutaneous candidiasis; GI, gastrointestinal; h, human; IA, invasive *Aspergillus*; IFN, interferon; IL, interleukin; KO, knockout; m, mouse; mLN, mesenteric lymph node; MR, mannose receptor; TNF, tumor necrosis factor; VVC, vulvovaginal candidiasis.

**Figure 3:**
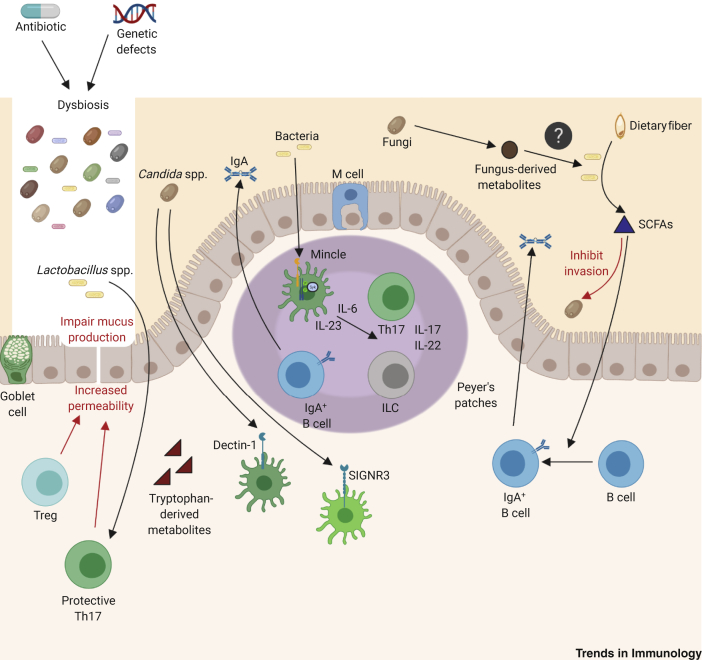
C-Type Lectin Receptors (CLRs) Can Shape Microbial Communities in the Gut and Maintain Homeostasis. Dysbiosis caused by antibiotic use or specific genetic defects (i.e., Dectin-1, CARD9, etc.) causes physiological and metabolic changes in the gut epithelium that increase susceptibility to inflammatory bowel disease (IBD) [96, 97, 98, 99, 100]. Tryptophan-derived metabolites produced by the gut microbiome and indoleamine 2,3-dioxygenase-expressing tolerogenic dendritic cells (DCs, not shown) support the differentiation of regulatory T cells (Tregs) and type 17 T helper cells (Th17s) that are important in protecting the gut from inflammatory disorders (red arrows) [50,83,103]. Short-chain fatty acid (SCFA) metabolites can inhibit *Candida* tissue invasion and promote colonization (red arrow) [57,58]. SCFAs also support plasma B cell differentiation, thus promoting antibody responses and decreasing susceptibility to infection [59]. CLRs expressed on intestinal DCs (i.e., Dectin-1 and SIGNR3) sense fungi present in the commensal microbiota, such as *Candida albicans*, and can influence the development of colitis [107,108]. DCs expressing Mincle play a central role in the Peyer’s patches and support Th17 cell differentiation by sensing mucosa-resident bacteria and producing interleukin (IL)-23 and IL-6 in a Syk-dependent manner [105]. The role of fungus-derived metabolites in gut homeostasis and other microbial communities is currently elusive (?). Abbreviation: ILC, innate lymphoid cell.

Of note, short-chain fatty acid (SCFA) metabolites, that are produced as fermentation products of dietary fiber by the bacterial component of the microbiota, can inhibit *Candida* tissue invasion and promote colonization [57,58] (Figure 3). SCFAs also support plasma B cell differentiation, promoting antibody responses and decreasing susceptibility to infection [59]. However, in humans, the role of B cells during fungal infections is only beginning to be revealed. For instance, individuals with agammaglobulinemia exhibit normal antifungal immunity [60].

Regarding other receptors, the mannose receptor (MR) was historically associated with the control of fungal infections owing to its ability to recognize high amounts of mannan in the outer fungal cell wall. MR does not contain an intracellular signaling domain and primarily acts as a homeostatic receptor which binds high mannosylated proteins removing them from the bloodstream [61]. Using human PBMCs, MR was recently shown to associate with FcRγ [62] (Figure 2). MR KO mice (B6.129P2-*Mrc1*^tm1Mnz^/J) displayed mild susceptibility to most fungal pathogens except pulmonary *C. neoformans* infection, in which increased lung fungal burdens and accelerated mortality were observed relative to WT mice [63]. Furthermore, *ex vivo* activation of CD4^+^ T cells from MR KO mice showed reduced proliferation in response to mannoproteins from *C. neoformans* compared with WT CD4^+^ T cells, implicating this receptor in T cell responses to this pathogen [63]. The apparent and unexpected lack of susceptibility to other fungal infections in MR KO mice has been speculated to be most likely due to the redundancy between receptors that sense mannans, including membrane-bound Dectin-2 and Mincle. Although the mechanisms are unclear, MR has been shown to be involved in the production of Th17 cytokines by human PBMCs challenged with *C. albicans* ex vivo [64].

Dectin-2 plays a central role in antifungal Th17 immunity. Unlike Dectin-1, absence of Dectin-2 does not affect innate antifungal immunity, but, in anti-Dectin-2 antibody-treated mice, *Candida*-specific IL-17 T cell responses are severely impaired relative to untreated mice [65], largely because of reduced production of IL-6, IL-1β, and IL-23 by DCs [66]. Dectin-2 KO mice (*Clec4n*^*−/−*^) also show increased susceptibility to systemic *Candida glabrata* infection, correlating with decreased production of IFN-γ and IL-10 relative to WT mice [67]. Accordingly, in response to pulmonary *C. neoformans* infection, Dectin-2 KO mice have significant increases in IL-4, IL-5, and IL-13 relative to WT mice [68]. Furthermore, Dectin-2 polymorphisms in humans are associated with increased susceptibility to pulmonary cryptococcosis relative to individuals harboring WT Dectin-2 [69]. Taken together, these studies suggest that the function of Dectin-2 in antifungal T cell immunity may be fungus species-specific.

Although ligand recognition and activation of CLRs promote a context for T cell differentiation that shapes protective Th1 and Th17 responses, several aspects remain unclear. A case in point is how distinct CLR output responses are sometimes nonredundant even though they all couple through CARD9 for signaling. For instance, Dectin-1 induces both Th1 and Th17 cells, whereas Dectin-2 is known to preferentially induce Th17 cells. *In vitro* activation of Dectin-1 with curdlan (a β-1,3-glucan polymer and a selective agonist of the Dectin-1 signaling pathway) induces transcription of the gene encoding IL-12p35 (a subunit of IL-12 that is usually under tight regulation that requires nucleosome remodeling by nuc-2); this appears to be consistent with a key role for Dectin-1 in conferring protection against systemic candidiasis, which relies heavily on Th1 immunity [70, 71, 72]. Dectin-1 also induces the expression of cytokines important for Th17 differentiation, including IL-6 and IL-23, in a Syk/CARD9 signaling-dependent manner (which included testing *Card9*^*−/−*^ mice) [73]. However, Dectin-2 preferentially induces the expression of IL-23p19 (a subunit of IL-23) and primarily promotes differentiation of Th17 immunity that is important for protection at mucosal tissues in mice [65]. This might also contribute to explaining why HIV-1-negative individuals with a Dectin-2 polymorphism show increased susceptibility to pulmonary cryptococcosis, but not to systemic infection, relative to individuals with WT Dectin-2 [69]. These differences in cytokine activation and T helper cell differentiation, even though both Dectin-1 and Dectin-2 couple to CARD9, may be partially explained by interactions with other pathogen recognition receptors and the activation of diverse signaling pathways. For example, biochemical assays have shown that Dectin-1 activation by *Fonsecaea monophora* (the causative agent of chromoblastomycosis, a chronic fungal skin infection) in human DCs *in vitro* activates interferon regulatory factor 1 (IRF1) which promotes transcription of the gene encoding IL-12p35, leading to protective Th1 and Th17 responses; however, simultaneous engagement of Mincle results in E3 ubiquitin-protein ligase Mdm2-dependent degradation of IRF1, which inhibits Dectin-1-mediated IL-12 production [74]. By contrast, Dectin-1 collaboration with Toll-like receptor TLR2 has been shown to result in amplification of Dectin-1-mediated signals in innate immune cells [75]. Understanding the outcome of these multiple receptor engagements and the effects within specific DC subsets will further our understanding of adaptive antifungal T cell immunity. Recently, Dectin-1 and Dectin-2 were both shown to be required for robust TNF production, compared with the individual receptors, following *C. albicans* infection in mouse models (as evidenced from the use of multi-CLR KO mice) [76]. Dectin-1 can also regulate the expression of other CLRs such as CD23 via nuclear factor of activated T-cells (NFAT) activation and JNK phosphorylation, as well as via Raf-1-mediated formation of inactive nuclear factor (NF)-κB (p65–RelB dimer); differential activation of these pathways may result in differential immune responses (as evidenced from phagocytosis and *in vitro* fungal killing assays using bone marrow-derived macrophages, BMDMs, from JNK1 KO vs WT mice) [77]. CLR negative regulation in mice, involving the E3 ubiquitin ligase, CBLB, which targets Dectin-1 and Dectin-2, has also been reported to inhibit downstream responses [78]. Thus, understanding the rules that govern these selective mechanisms is required if we are to fully understand how CLRs shape adaptive antifungal immunity.

### Treg Helper Cells

Tregs are a class of T helper cells that regulate immune responses. Different subsets of Tregs have been reported: for example, these include thymus-derived natural Tregs (nTregs), that are characterized by the expression of Helios and FoxP3, and peripherally induced Tregs (iTregs), Foxp3^+^ or Foxp3^−^, that secrete anti-inflammatory cytokines including TGF-β, IL-10, and/or IL-35 [79]. How these subsets are generated in response to fungal infections remains unclear, especially regarding iTregs and their functions. In mice challenged systemically with *C. albicans*, FoxP3^+^ Tregs were shown to promote immune responses, including the induction of IgA which is important for protecting the mucosae [80] (Figure 1). Others have shown that stimulation of human PBMCs with *A. fumigatus ex vivo* elicits a predominant Treg response without an increase in effector T cells, highlighting the tolerogenic potential of Tregs in humans and the putative role of these cells in dampening immune responses to other airborne fungal species [81,82]. This notion is supported by findings in mice (using genetic models) showing that, during early stages following lung exposure to *A. fumigatus* conidia, CD4^+^CD25^+^ Tregs expand and limit neutrophil inflammation in lungs via IL-10 and CTLA-4 [83]. At a later adaptive phase, Tregs expressing IL-10 and TGF-β were induced in a mechanism involving a tolerogenic DC program mediated by indoleamine 2,3-dioxygenase (IDO), and these Tregs can inhibit Th2 cells, preventing fungal asthma in the mice [83]. In another study, during experimental pulmonary cryptococcal infection in mice, Foxp3^+^ Treg cells were shown to suppress the detrimental effects of Th2 cells (that are associated with a nonprotective outcome) *in vivo* [84,85]. By contrast, in a systemic *C. albicans* intravenous infection mouse model, failure to control fungal burdens involved *Candida* antigen-driven expansion of both Foxp3^+^ nTreg cells and Foxp3^−^ iTregs (converted from Rorγt^+^ Th17 cells); Foxp3^+^ cells enhanced Th17 responses and inhibited Th1 and Th2 cells relative to control mice [86]. Indeed, depletion of Foxp3^+^ in Foxp3(hCD2) reporter mice led to exacerbated fungal burden and inflammatory renal disease [86]. Thus, given their influence in immune modulation, future work should focus on investigating the roles and functions of various Treg subsets during fungal commensalism and pathogenesis at different tissue sites.

### Impact of Fungal Morphology

Morphogenic transitions occur in some fungi in response to environmental cues or host-derived stimuli. Dimorphic fungi including *C. albicans* switch between yeast, pseudohyphal, and hyphal forms in response to changes in temperature, nutrient availability, or pH. Yeast morphology is generally associated with commensalism and dissemination, whereas hyphae are required for tissue invasion and pseudohyphae are predominantly found in systemic organs [87]. These morphological changes affect recognition by immune cells and the subsequent induction of immunity (Figure 2). For instance, depletion of the micronutrient zinc drives the formation of enlarged *C. albicans* yeast cells, named Goliath cells, that exhibit remarkable differences in pathogen-associated molecular pattern (PAMP) exposure and cell adhesion [88]. In addition, antifungal drug therapy, such as with caspofungin, leads to alterations in fungal cell walls that affect β-glucan exposure and subsequent recognition and activation of immune cells [89]. Of note, fungal morphology not only affects pathogenicity but can also determine T helper cell differentiation. For instance, in the skin, filamentous forms of *C. albicans* induce a Th1 response whereas yeasts preferentially promote a Th17 response through Dectin-1 engagement on Langerhans cells (skin cells) [90]. Contrary to these results, *in vitro*, *C. albicans* yeast cells were shown to induce human Th1 cells via IL-12 production, while *C. albicans* hyphae selectively induced Th17 responses via IL-23 production [91]. Moreover, *C. neoformans* can transition from haploid yeast to polyploid Titan cells (>10 μm) in the murine lung [92]. Unlike the well-studied yeast form, the type of T cell responses induced by Titan cells remains unclear. The scant data on the impact of fungal morphological changes on immune recognition by CLRs, as well as the new repertoire of antigenic peptides that are generated, are areas that clearly require further investigation.

## The Mycobiome

Fungi colonize various ecological niches including mammalian oral, urogenital, placental, skin, airway, and gut mucosae, and their interactions with the host significantly impact on health and disease. Although not fully understood, recent studies suggest that fungal colonization during early life impacts on the development and maturation of the host immune system and subsequent protection or susceptibility to disease [93]. For instance, colonization with fungal species such as *Candida*, *Rhodotorula*, *Penicillium*, *Aspergillus*, and *Alternaria* correlates with the risk of developing childhood **atopy** and asthma driven by Th2 immunity [94,95]. By contrast, *Cladosporium* exposure can protect against the development of allergy [95]. Of relevance, alterations of the human gut mycobiota caused by antimicrobial use can contribute to the development of fungus-induced allergy, IBD, and systemic fungal infections [96, 97, 98, 99, 100] (Figure 3). For instance, antibiotic-dependent intestinal expansion of the commensal fungus *Wallemia mellicola* enhances the severity of allergic airway disease in mice [96]. Furthermore, in mice, treatment with antibiotics can lead to overgrowth of *C. albicans* in the gastrointestinal tract and increase allergic airway responses following intranasal inoculation with *A. fumigatus* [101]. Supporting these data, during pulmonary *A. fumigatus* infection, oral antibiotic treatment was shown to reduce Th17 functional responses, while increasing Th2 responses in the mouse lung, relative to untreated mice [102].

Recent developments have linked gut fungal Th1/Th17 immune responses with protection to systemic infections. For instance, intestinal colonization by *C. albicans* in mice can protect the host not only from distal fungus-associated infections such as disseminated *C. albicans* and pulmonary *A. fumigatus* but also from other pathogens such as *Staphylococcus aureus* and *Pseudomonas aeruginosa* [103,104]. In humans, protective Th17 cells are predominantly driven by gut *C. albicans* which contribute to preventing pathogenesis by these opportunistic commensal fungi [103]. As previously discussed, crossreactive *Candida*-specific Th17 cells can be triggered to expand by other fungi such as airborne *Aspergillus* spores, resulting in detrimental lung inflammation in humans and mice [51]. These findings highlight the fine line between the protective and pathogenic potential of T helper cells and their association with gut microbes. Nevertheless, induction of cellular systemic immunity through modulation of the mucosal mycobiome is an attractive approach to tackling infection. To fully explore this, however, it will be necessary to understand the mechanisms that modulate antifungal T cell maintenance and memory development, as well as the regulatory mechanisms involved.

Microbial communities within the gut interact with each other and induce activation of immune cells to maintain homeostasis and prevent invasion of opportunistic commensal organisms. Mincle was shown to sense mucosa-resident bacteria and to promote intestinal barrier integrity through the activation of several responses, including upregulation of IL-6, IL-23p19, as well as via activation of, for example, group 3 innate lymphoid cells (ILC3s), the induction of Th17 cells, and secretion of IL-17 and IL-22 in mice (using various genetic models) [105]. In addition, failure to appropriately sense commensal gut fungi through the loss of molecules such as Dectin-1, the non-CLR ephrin type-A receptor 2 (EphA2, expressed on oral epithelium), and SIGNR3 (the closest murine homolog of the human DC-SIGN) has been linked to aggravated disease outcomes (e.g., colitis) as well as to overgrowth of fungi in the gut in both mice and humans [106, 107, 108] (Figure 3).

From another angle, the CLR–Syk–CARD9 signaling cascade is essential for controlling opportunistic commensal fungi in the gut. A recent study in both mice and humans demonstrated that CLR-expressing colon-resident chemokine receptor CX3CR1^+^ mononuclear phagocytes could recognize fungi and control gut antifungal T cell immunity in a Syk-dependent manner (e.g., as evidenced by genetic ablation of CX3CR1^+^ mononuclear phagocytes in mice, and a missense mutation in CXCR1 in Crohn’s disease patients) [109]. Furthermore, *Candida tropicalis*, among other fungal species, was shown to protect against colon cancer through CARD9 signaling in intestinal myeloid cells (myeloid-specific deletion of *Card9* and *Syk* in mouse models of colitis and tumorigenesis) [110,111]. Moreover, Card9-deficient mice lack several microbiota constituents, including *Lactobacillus* spp., and are more susceptible to colonization with *Candida* in the gastrointestinal (GI) tract than are WT mice [112]. Furthermore, these mice fail to metabolize tryptophan into **aryl-hydrocarbon receptor (AhR)** ligands, metabolites that may be crucial for Th17 and Treg cell induction [112] (Figure 3). Of note, in humans, rare SNPs in *CARD9*, that lead to truncated or missense mutations in the CARD9 protein (C-terminal tail), have been linked to increased susceptibility to fungal infections and IBD, respectively. Indeed, Crohn’s disease patients with CARD9 SNPs have been reported to exhibit increased colonization with *Malassezia* relative to WT CARD9 SNPs – findings that were replicated in mouse models [113]. Although mostly still unclear, taken together, these data suggest that highly organized mechanisms regulate host–mycobiome interactions in health and disease.

## Concluding Remarks

CLRs have been studied in detail in the context of innate immunity, and recent studies have also defined an essential role for CLRs in the context of antifungal adaptive T cell immunity. However, several key questions remain unanswered (see Outstanding Questions). Many of the studies linking CLRs to T cell immunity have focused on the effects of DC-derived cytokines and their influence on T cell differentiation. At present, little is known about other mechanisms that shape T cell immunity during fungal infections, such as the role of co-stimulation and ensuing signaling pathways (Figure 2). Recent data in mice show, for example, that Dectin-1 stimulation with curdlan upregulates co-stimulatory molecules such as OX40L and TNFSF15 which are involved in the regulation of T helper cell differentiation [114]. Indeed, Dectin-1, Dectin-2, and Mincle can activate the induction of various co-stimulatory molecules including CD40 and CD86 in mice [115, 116, 117]. In addition, CLRs can also stimulate innate immune memory, a recently described general concept whereby innate immune cells undergo epigenetic modifications that can provide cross-species immunological memory resulting in altered responses upon rechallenge [118]. How all these systems are linked remains elusive, but this is certainly an area of investigation that merits attention to develop improved candidate therapeutics against fungal and other infections.

CLR expression is commonly linked to innate cells; however, detailed expression patterns of CLRs in different APCs, and the type of immune responses they mediate upon recognition of fungi during conditions of commensalism versus pathogenesis, remain to be fully explored. Understanding the interactions between fungi and other microorganisms such as bacteria, viruses, and parasites will also prove invaluable for understanding how communities shape local and distal immune responses. Further insight into the effects of these interactions on immune responses will be important for the development of therapeutics against polymicrobial interactions. In the future, application of cutting-edge technologies in metagenomics, transcriptomics, proteomics, and metabolomics may help to decipher many of these interaction networks and provide valuable insight into the impact of fungi and CLRs on homeostasis and immunity.

Outstanding Questions How are signals from different CLRs regulated and integrated following fungal recognition?

What are the expression patterns of CLRs across different APCs such as tissue-resident dendritic cells? Do these expression patterns allow C-type lectins to sense differences between commensal and pathogenic fungi? Or do they provide tissue specific responses? Do these change during inflammation?

CLRs have been implicated in epigenetic mechanisms that regulate innate immune memory: how does this integrate with their role in the development of adaptive immunity?

Is there a repertoire of co-stimulatory molecules that are regulated by CLRs, and how does this influence adaptive immune responses?

How are unwanted Th17 responses terminated while maintaining their protective effects? Functional plasticity of Th17 cells creates a fine line between beneficial and detrimental effects. Can this be understood in enough detail to successfully produce immunotherapies?

How does C-type lectin-mediated recognition of fungi influence adaptive immune responses to other microbes such as viruses, parasites, and bacteria?

## Glossary

**Agonist**: a chemical compound that mimics the action of another substance (such as a receptor or ligand) to produce a similar response.
**Antagonist**: a chemical compound that blocks the action of an agonist (including receptor–ligand interactions) and thus inhibits the response.
**APC maturation**: following phagocytosis of a foreign pathogen, APCs begin maturation whereby they lose the ability to phagocytose further pathogens, and instead load antigens onto MHC molecules for surface expression, upregulate cosignaling molecules, and start to release chemokines and cytokines.
**Aryl-hydrocarbon receptor (AhR)**: a ligand-induced transcription factor that regulates the expression of a diverse set of genes, including those involved in metabolism and immune regulation.
**Atopy**: genetic predisposition to developing allergic diseases such as asthma and eczema.
**Autoantibodies**: antibodies produced by B cells that are directed against one or more self-antigens. They commonly contribute to the development of autoimmune diseases.
**Commensal fungi**: fungi which reside in the natural flora of the human body; these coevolved to benefit from living within or on the host but neither harm nor benefit the host.
**Co-stimulatory molecules**: a diverse range of molecules that provide either stimulatory or inhibitory signals; for example, the B7:CD28 and TNF superfamilies. They provide non antigen-specific signals during T and B cell activation and induce co-stimulatory signaling pathways.
**Homeostasis**: regulation of normal physiological conditions; recognition of foreign antigens promotes immune cells to restore the environment to normality.
**Hyper-IgE syndrome**: a primary immunodeficiency caused by mutations in *STAT3*; patients present with high serum concentrations of IgE and defective Th17 immunity, and exhibit recurrent skin and pulmonary infections and eczema.
**Inflammatory bowel disease (IBD)**: a group of inflammatory conditions that affect the colon and small intestine, namely ulcerative colitis and Crohn’s disease.
**Major histocompatibility complex (MHC)**: molecules that bind peptide fragments of exogenous and endogenous origin for presentation to T cell receptors. MHC-I molecules present antigen to CD8^+^ T cells, whereas MHC-II molecules present antigen to CD4^+^ T cells.
**Opportunistic pathogens**: under normal conditions these organisms are nonpathogenic; host-derived and/or environmental alterations, such as host immunodeficiency or pathogen acquisition of virulent genes, can lead them to pathogenesis and infection.
**Pulmonary alveolar proteinosis**: a rare lung disease caused by mutations in surfactant proteins or GM-CSF; the disease is characterized by ineffective clearance of surfactant in lung alveoli by alveolar macrophages.
**Rag1 KO mice (*Rag1***^***−/−***^**)**: KO mice that lack Rag1 (an enzyme important for somatic recombination of antigen receptor genes during T cell and B cell maturation that is important for generating of the antigen receptor repertoire), thus being devoid of mature T and B cells, and are deficient in adaptive immunity.
